# Induction of labour in pre-eclamptic women: a randomised trial comparing the Foley balloon catheter with oral misoprostol

**DOI:** 10.1186/1471-2393-14-308

**Published:** 2014-09-05

**Authors:** Hillary Bracken, Shuchita Mundle, Brian Faragher, Thomas Easterling, Alan Haycox, Mark Turner, Zarko Alfirevic, Beverly Winikoff, Andrew Weeks

**Affiliations:** Gynuity Health Projects, 15 East 26th Street, Suite 801, New York, NY 10010 USA; Department of Obstetrics & Gynecology, Government Medical College, Nagpur, 440003 India; Medical Statistics, LSTM Clinical Group, Liverpool School of Tropical Medicine, Pembroke Place, Liverpool L3 5QA UK; Department of Obstetrics and Gynecology, University of Washington, Seattle, Washington 98195 USA; Department of Women’s and Children’s Health, University of Liverpool Management School, Chatham Street, Liverpool, L69 7ZH UK; Department of Women’s and Children’s Health, University of Liverpool, Liverpool Women’s Hospital, Crown Street, Liverpool, L8 7SS UK

**Keywords:** Preeclampsia, Misoprostol, Foley catheter, Induction of labor

## Abstract

**Background:**

Between 40,000 and 80,000 pregnant women die annually from pre-eclampsia and eclampsia. Although magnesium sulphate and anti-hypertensive therapies can reduce the morbidity and mortality associated with pre-eclampsia, the only cure comes with delivery. Prompt delivery of the baby, preferably by vaginal route, is vital in order to achieve good maternal and neonatal outcomes. Induction of labour is therefore a critical intervention in order to prevent morbidity to both mother and baby. Two low cost interventions – oral misoprostol tablets and transcervical Foley catheterization – are already used by some in low resource settings, but their relative risks and benefits are not known. The trial will compare the risks, benefits, and trade-offs in efficacy, safety, acceptability and cost of misoprostol and Foley catheter for induction in women with preeclampsia or uncontrolled hypertension.

**Methods/Design:**

A total of 602 women with an ongoing pregnancy with a live fetus requiring delivery because of pre-eclampsia or uncontrolled hypertension will be randomly assigned to labor induction with a transcervical Foley catheter or oral misoprostol 25 micrograms. Women will be recruited at two hospitals in Nagpur, India. The misoprostol group will receive oral misoprostol 25 microgram every 2 hours for a maximum of 12 doses or until active labor commences. The Foley group will undergo induction using a Foley catheter (silicone, size 18 F with 30 ml balloon) which will remain until active labor starts, the Foley catheter falls out, or 12 hours have elapsed. The primary outcome will be the attainment of vaginal delivery within 24 hours. Providers administering the treatment and those assessing the outcomes will not be blinded to group assignment.

**Trial registration:**

NCT01801410 (ClinicalTrials.gov).

## Background

Between 40,000 and 80,000 pregnant women die annually from pre-eclampsia and eclampsia [1]. Magnesium sulphate and anti-hypertensive therapies can reduce the morbidity and mortality associated with pre-eclampsia [2, 3]. The only cure, however, comes with delivery. Prompt delivery of the baby, preferably by vaginal route, is vital in order to achieve good maternal and neonatal outcomes. Induction of labour is therefore a critical intervention in order to prevent morbidity and mortality to both mother and baby. Two low cost interventions – oral misoprostol tablets and transcervical Foley catheterization – are already used by some in low resource settings, but their relative risks and benefits are not known. These interventions could optimize the care pathway for women needing induction of labour. This is especially important in low resource settings where improvement is most needed and the potential to reduce the maternal and neonatal mortality and morbidity is the greatest. The ideal induction agent would result in a relatively short induction to delivery interval without risk to fetus and with low rates of emergency caesarean section. The induction to delivery interval is especially important in pre-eclampsia and eclampsia where the condition may deteriorate rapidly.

Misoprostol, the orally active and heat stable prostaglandin E1 analogue, has been used for labour induction for nearly 20 years. Following years of debate about appropriate dosage, it appears that the use of oral misoprostol 25 microgram is optimal [4, 5]. A systematic review of low dose oral misoprostol included two trials in which the 25 microgram dose of oral and vaginal misoprostol were compared. It found that “women using oral misoprostol were significantly less likely to experience uterine hyperstimulation with fetal heart rate changes (2% compared with 13%; RR 0.19, 95% CI 0.08-0.46), but there were no significant differences in other outcomes [6]”. The Cochrane review concluded that “Given that safety is the primary concern, the oral regimens are recommended over vaginal regimens. This is especially important in situations where the risk of ascending infection is high and the lack of staff means that women cannot be intensely monitored [4]”. Oral misoprostol is very effective but still has a hyperstimulation rate of 6% [6], potentially causing hypoxic damage to the fetus. The risk of hypoxic damage is increased in pre-eclampsia, where babies may be born prematurely or affected by intrauterine growth restriction. In low resource settings, where access to intrapartum fetal monitoring may be limited, avoidance of hyperstimulation, which may go undetected, is critical.

Alternative low cost methods have been sought that can induce labour with potentially less risk to the fetus. Such an agent may be the Foley catheter. Although usually used for bladder drainage, these catheters can be inserted into the cervix and held there by inflating the balloon. The catheter is then placed on gentle traction by strapping it to the mother’s thigh and left for 12 hours or until it falls out through the cervical os. Induction with the Foley balloon catheter appears to be as effective as current standard methods, but with lower rates of uterine hyperstimulation and better fetal outcomes [7, 8]. However, the majority of the studies (22/30) were conducted in Western settings. Little is known about the rates and risks of infection in low resource settings.

Low-dose misoprostol appears to be an effective alternative for labor induction although inductions with prostaglandins, including misoprostol, are sometimes associated with uterine hyperstimulation and consequent fetal hypoxia. The Foley balloon induction method may have considerable safety benefits for the fetus, although there is conflicting evidence as to its effect on the speed of induction. Both methods are promising, but the relative risks and benefits of the two methods for labor induction among women with pre-eclampsia in low resource settings has yet to be established in a large, randomized trial. We will conduct a multicentre randomized trial comparing misoprostol treatment with Foley balloon induction for labor induction in women with preeclampsia or uncontrolled hypertension. This study will identify the risk, benefits and trade-offs in efficacy, safety, acceptability and cost of these two low cost induction methods.

## Methods

This pragmatic, open-label, randomised control trial will enroll patients seeking care for preeclampsia at Government Medical College and Daga Women’s Hospital, Nagpur. Women with an ongoing pregnancy with a live fetus in whom the decision has been made to induce vaginal delivery because of preeclampsia or uncontrolled hypertension will be included. Women will be excluded if they are less than 18 years old; have previously undergone a caesarean section; present with a multiple pregnancy, ruptured membranes, or chorioamnionitis; or report a history of allergy to misoprostol. All subjects will provide written and oral informed consent and have the informed consent process video recorded as required by Indian regulations. This protocol was approved by the Liverpool School of Tropical Medicine Research Ethics Committee (Approval number 12.26) and the Ethics Committee at Government Medical College, Nagpur and will be conducted in compliance with the Helsinki Declaration.

### Randomisation

**Figure 1:**
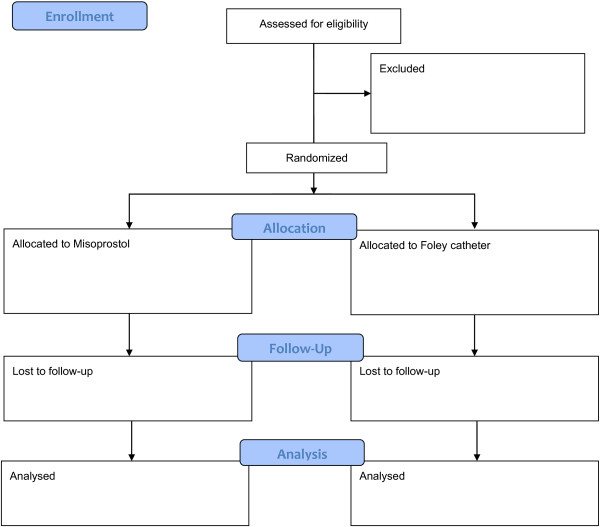
**Consort flow diagram.**

At enrolment, the medical history will be assessed and a physical exam will be performed (Figure 1). Women will be randomised independently using a computerised random number generator; the randomisation will be stratified by centre using a randomly determined block size. The group assignment will be indicated in a card sealed in sequentially numbered opaque envelopes. The group assignment will be revealed immediately prior to the start of the induction. The participants, providers, and those assessing outcomes will not be blinded to the group assignment.

Women in the mechanical group will undergo induction using a transcervical Foley catheter (silicone, size 18 F with 30 ml balloon) which will remain in place until active labour starts, the Foley catheter falls out, or 12 hours have elapsed. If the Foley catheter falls out within 12 hours, membranes will be ruptured and/or oxytocin infusion started. If the Foley catheter does not fall out within 12 hours, it will be removed at 12 hours and oxytocin commenced with an artificial rupture of membranes when possible. If labour has still not commenced after 24 hours, the woman will be deemed to have a ’failed induction’ and the decision on further management will be made by the clinical team. Their choice of method could include the use of misoprostol, repeat Foley catheter, dinoprostone, caesarean section or delay as deemed appropriate.

Women in the misoprostol group will be induced using oral misoprostol tablets (25 micrograms, Cipla Ltd, Mumbai) every 2 hours for a maximum of 12 doses or until active labour commences. In primigravid women, if contractions have not commenced after 2 doses, the dosage may be increased to 50 micrograms every 2 hours. Once in labour (regular painful contractions with a cervical dilatation of at least 4 cm) no more misoprostol will be used and artificial membrane rupture and/or oxytocin infusion will be used as clinically indicated. If labour has still not commenced after 24 hours, the woman will be deemed to have a ‘failed induction’ and the decision on further management will be made by the clinical team. Their choice of method could include the use of misoprostol, repeat Foley catheter, dinoprostone, caesarean section or delay as deemed appropriate.

During the labor induction, women will be monitored by research clinicians every two hours. The use of any additional medications, cervical dilation and Bishop score will be recorded, as appropriate.

Women will be asked to assess their expectations prior to the induction process. Then, within 48 hours of delivery and prior to discharge, women will be interviewed by study staff using a short questionnaire. The questionnaire will assess their opinions about their experience and issues surrounding the induction (including time to induction), their satisfaction with the actual labour, and their perspectives on the two medical technologies. Women will be asked to rate their overall experience on a categorical five-point scale from ‘very unsatisfied’ to ‘very satisfied’ and compare this to their actual experience, with a focus on what they would like to change in any future induction. Care providers’ (nurses and doctors) opinions will also be collected in a separate provider survey (or focus group discussion where appropriate) after the conclusion of the trial.

Participants with severe pre-eclampsia will receive magnesium sulfate and anti-hypertensives as per local protocols.

### Study outcomes

The primary outcome will be the attainment of vaginal delivery within 24 hours. Vaginal delivery within 24 hours is the standard primary outcome suggested in the Cochrane Collaboration induction of labour generic protocol [5, 6]. It combines an acceptable time period for the induction process as well as the combined fetal and maternal safety outcome of need for caesarean section. Caesarean section also poses a serious risk to maternal health in the context of the setting and disease.

Secondary outcomes will assess the success of the induction process, neonatal morbidity and mortality, and maternal mortality and morbidity. Measures of efficacy of the induction process will include the induction to delivery interval (in vaginal deliveries, caesarean sections and all deliveries), the proportion of women in each group with vaginal deliveries within 12 hours, with cervix unchanged at 12 and 24 hours, and with a need for oxytocin augmentation. Measures of maternal complications will include uterine tachysystole (defined as over 5 contractions in 10 minutes), uterine hypertonus (defined as a single contraction lasting over 2 minutes), caesarean section, uterine rupture, instrumental vaginal delivery, severe hypertension and HELLP Syndrome, maternal vomiting, maternal diarrhoea, fever, antibiotic use, and postpartum haemorrhage. Data on the following fetal-neonatal complications will also be collected: meconium-stained liquor, Apgar score less than seven at five minutes, neonatal intensive care unit admission, seizures, birth asphyxia, and stillbirth.

### Statistical analysis

The primary outcome will be vaginal birth within 24 hours. The rate of vaginal delivery within 24 hours in the Foley catheter group is estimated at 41%. This rate is taken from the study by Pennell [9] which compared a Foley catheter for 12 hours with vaginal misoprostol and found a rate of vaginal delivery in the Foley group within 24 hours of 53/110 (48%) [9]. Only two other studies were identified that used the same induction protocol and outcomes as planned for this study (i.e. a 12 hour Foley under traction and an outcome of vaginal birth within 24 hours). In Yuen [10] the vaginal delivery rate within 24 hours was 15/36 (42%), and in Owolabi [11] the rate was 17/60 (28%) [10, 11]. The combined rate from these 3 studies is 85/206 (41%).

In the Cochrane review of oral misoprostol 25 microgram versus PGE2 which included 5 trials, the vaginal delivery rate within 24 hours in the 25 microgram oral misoprostol group was 654/1089 (60%) [4]. The vaginal delivery rate in this study is likely to be increased because there will be little intrapartum fetal monitoring, a factor that is known to increase caesarean section rates. However, more women will be having labor induced preterm (with more unfavourable Bishop scores) – a factor that also increases caesarean section rates. Our estimated rate of vaginal delivery within 24 hours in the misoprostol group is therefore 60%.

The null hypothesis is that there is no difference in the rate of vaginal delivery within 24 hours in those induced with misoprostol and those induced with a Foley catheter. This hypothesis will be rejected if there is a 33% difference in the outcomes with the two methods. To detect an absolute increase from 41% to 55% with 90% power (two-sided α = 0.05) will require a sample of 602 women. If the vaginal birth rate in the Foley group is higher, then the study will have more power. As stated previously, we do not anticipate any loss to follow-up.

### Proposed analyses

Logistic, Poisson and negative binomial regression models will be used as appropriate to estimate effect size (difference in vaginal delivery rates) with 95% confidence intervals; adjustments will be made for important confounding variables and covariates. The primary analysis will be done according to intention to treat. If indicated, a secondary, per protocol analysis will be performed. Similar regression models will be used to compare the two study groups with respect to other important (secondary) variables. Subgroup analyses will be parity (nulliparous or not), site of delivery, Bishop score (≥6 or less), and perceived fetal viability (determined by the provider at enrolment).

We will conduct an economic evaluation to assess patterns and levels of resource utilisation associated with the two induction techniques. This analysis will enable any variations in resource use between the two treatment groups to be identified, measured and valued and facilitate the linking of patterns/costs of resource use to maternal and child health outcomes and other agreed measures of ‘effectiveness’ and ‘success’.

An interim analysis will be conducted after one year of recruitment, or when data are available from the first 300 women. The data will be reviewed by an independent Data Monitoring Committee. Further interim analyses are not planned, but may be requested by the Data Monitoring Committee. No interim analyses for effectiveness or futility are planned.

## References

[1] Khan KS, Wojdyla D, Say L, Gülmezoglu AM, Van Look PF (2006). WHO analysis of causes of maternal death: a systematic review. Lancet.

[2] Altman D, Carroli G, Duley L, Farrell B, Moodley J, Neilson J, Smith D (2002). Magpie Trial Collaboration Group. Do women with pre-eclampsia, and their babies, benefitfrom magnesium sulphate? The Magpie Trial: a randomised placebo-controlled trial. Lancet.

[3] Duley L, Meher S, Jones L (2013). Drugs for treatment of very high blood pressure during pregnancy. Cochrane Database Syst Rev.

[4] Alfirevic Z, Weeks A: **Oral misoprostol for induction of labour.** *Cochrane Database Syst Rev* 2006, (2)**:**CD001338. doi:10.1002/14651858.CD001338.pub2 10.1002/14651858.CD001338.pub2 16625542

[5] Hofmeyr GJ, Gülmezoglu AM, Pileggi C: **Vaginal misoprostol for cervical ripening and induction of labour.** *Cochrane Database Syst Rev* 2010, (10)**:**CD000941. doi:10.1002/14651858.CD000941.pub2 10.1002/14651858.CD000941.pub2 PMC7061246 20927722

[6] Kundodyiwa TW, Alfirevic Z, Weeks AD (2009). Low-dose oral misoprostol for induction of labor: a systematic review. Obstet Gynecol.

[7] Jozwiak M, Oude Rengerink K, Benthem M, van Beek E, Dijksterhuis MG, de Graaf IM, van Huizen ME, Oudijk MA, Papatsonis DN, Perquin DA, Porath M, van der Post JA, Rijnders RJ, Scheepers HC, Spaanderman ME, van Pampus MG, de Leeuw JW, Mol BW, Bloemenkamp KW, PROBAAT Study Group (2011). Foley catheter versus vaginal prostaglandin E2 gel for induction of labour at term (PROBAAT trial): an open-label, randomised controlled trial. Lancet.

[8] Jozwiak M, Bloemenkamp KWM, Kelly AJ, Mol BWJ, Irion O, Boulvain M: **Mechanical methods for induction of labour.** *Cochrane Database Syst Rev* 2012, (3)**:**CD001233. doi:10.1002/14651858.CD001233.pub2 10.1002/14651858.CD001233.pub2 22419277

[9] Pennell CE, Henderson JJ, O'Neill MJ, McCleery S, Doherty DA, Dickinson JE (2009). Induction of labour in nulliparous women with an unfavourable cervix: a randomised controlled trial comparing double and single balloon catheters and PGE2 gel. BJOG.

[10] Yuen PM, Pang HYY, Chung T, Chang A (1996). Cervical ripening before induction of labour in patients with an unfavourable cervix: a comparative randomized study of the Atad ripener device, prostaglandin E2 vaginal pessary, and prostaglandin E2 intracervical gel. Aus NZ J Obstet Gynecol.

[11] Owolabi AT, Kuti O, Ogunlola IO (2005). Randomised trial of intravaginal misoprostol and intracervical Foley catheter for cervical ripening and induction of labour. J Obstet Gynaecol.

[CR12] The pre-publication history for this paper can be accessed here: http://www.biomedcentral.com/1471-2393/14/308/prepub

